# CRISPR–Cas-mediated transcriptional control and epi-mutagenesis

**DOI:** 10.1093/plphys/kiac033

**Published:** 2022-02-03

**Authors:** Jason Gardiner, Basudev Ghoshal, Ming Wang, Steven E Jacobsen

**Affiliations:** Department of Molecular, Cell and Developmental Biology, University of California at Los Angeles, Los Angeles, California, USA; Department of Molecular, Cell and Developmental Biology, University of California at Los Angeles, Los Angeles, California, USA; Department of Molecular, Cell and Developmental Biology, University of California at Los Angeles, Los Angeles, California, USA; Department of Molecular, Cell and Developmental Biology, University of California at Los Angeles, Los Angeles, California, USA; Howard Hughes Medical Institute (HHMI), UCLA, Los Angeles, California, USA

## Abstract

Tools for sequence-specific DNA binding have opened the door to new approaches in investigating fundamental questions in biology and crop development. While there are several platforms to choose from, many of the recent advances in sequence-specific targeting tools are focused on developing Clustered Regularly Interspaced Short Palindromic Repeats- CRISPR Associated (CRISPR-Cas)-based systems. Using a catalytically inactive Cas protein (dCas), this system can act as a vector for different modular catalytic domains (effector domains) to control a gene's expression or alter epigenetic marks such as DNA methylation. Recent trends in developing CRISPR-dCas systems include creating versions that can target multiple copies of effector domains to a single site, targeting epigenetic changes that, in some cases, can be inherited to the next generation in the absence of the targeting construct, and combining effector domains and targeting strategies to create synergies that increase the functionality or efficiency of the system. This review summarizes and compares DNA targeting technologies, the effector domains used to target transcriptional control and epi-mutagenesis, and the different CRISPR-dCas systems used in plants.

## Introduction

Over the past three decades, the development of tools that can bind to DNA in a sequence-specific manner has led to technologies that can specifically target and regulate gene transcription and epi-mutagenesis. Due to its ease of use, in the most recent wave of developments, tools based on the Clustered Regularly Interspaced Short Palindromic Repeats-CRISPR-associated system (CRISPR–Cas) using a catalytically inactive Cas protein have come to the forefront with an array of attachments allowing for the targeted transcriptional control or epi-mutagenesis of a specific locus; pushing the functionality of CRISPR–Cas beyond gene editing.

These constructs rely on either the direct recruitment of basal transcription machinery or the targeting of epigenetic factors to manipulate transcription of nearby genes. While the recruitment of basal transcription machinery requires the presence of the targeting construct, changes in DNA methylation can in some cases be mitotically and meiotically inherited allowing for this targeted epi-mutagenesis to be maintained in the following generations in the absence of the targeting construct (Johnson et al., 2014; Gallego-Bartolomé et al., 2018; Papikian et al., 2019). The heritability of DNA methylation is well documented at a few loci; however, further work is needed to understand how frequently methylation changes induced in one generation can be stably inherited into the next (Lloyd and Lister, 2021). Furthermore, most other plant epigenetic marks are likely not inherited from generation to generation and are either reset during reproductive development or change dynamically along with changes in gene expression. As we continue to develop targeting systems through the addition of different modular catalytic domains (effector domains) capable of manipulating specific epigenetic marks, the function and heritability of these marks can be more easily explored. The expansion of this toolkit will offer a selection of tools that can create changes that are or are not mitotically or meiotically inherited that can be selected based on the end goal.

In plants, targeted manipulation of transcriptional control and epimutagenesis have already been used to increase resistance to drought, manipulate plant developmental phenotypes, increase our understanding of interactions between essential proteins, and further our understanding on how plants add, maintain and use DNA methylation (Johnson et al., 2014; Harris et al., 2018; Gallego-Bartolomé et al., 2019; Papikian et al., 2019; Roca Paixão et al., 2019; Ichino et al., 2021; Lee et al., 2021; Leydon et al., 2021; Liu et al., 2021; Tang et al., 2021; Xue et al., 2021). As we continue to develop these tools by improving their efficiency and expanding the available effector domains, more possible applications in both research and industry arise. Fundamental questions that once relied on generalities and correlations resulting from mutations, stress induction, or chemical treatments can instead be addressed using targeted studies providing causative data revealing locus-specific function. While many of these tools have been demonstrated or developed in animals, plants provide an exciting platform for the further development and application of these tools both from a research and an agronomic perspective.

In this review, we will cover the recent discoveries and advancements in targeted transcriptional control and epi-mutagenesis using CRISPR–dCas-based targeting technologies in plants.

## Epigenetics and plant gene expression

**Figure 1:**
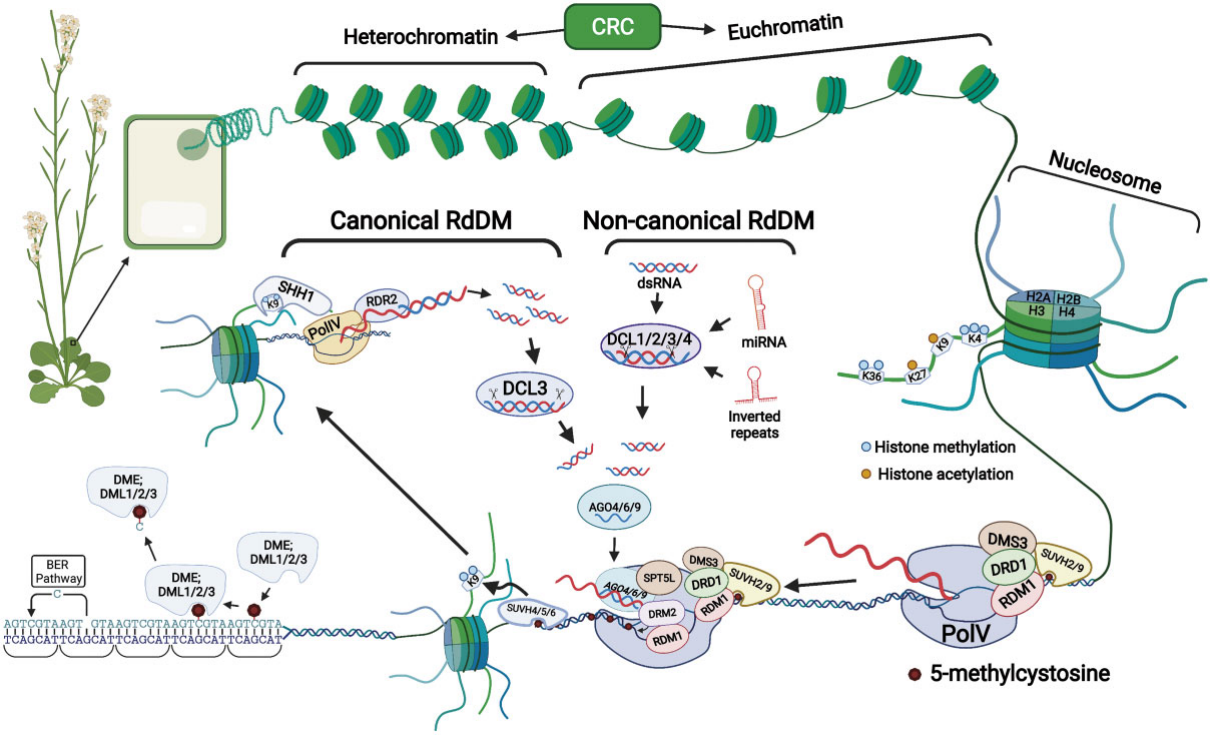
Overview of histone and DNA epigenetic modifications. Heterochromatin and euchromatin represent a more compact or less compact chromatin status, respectively, that can be manipulated by CRCs. The fundamental unit of chromatin is the nucleosome, which is composed of histones wrapped with DNA. Both histone tails and DNA cytosines can be epigenetically modified. Histone tail modifications: histone tails can be modified by various epigenetic marks, including methylation, acetylation, phosphorylation, SUMOylation, etc. Histone epigenetic marks are associated with transcriptional gene regulation. For example, histone acetylation and trimethylation of H3 Lysine 4 (H3K4me3) and H3K36me3 are associated with transcriptional activation. De novo DNA cytosine methylation: Cytosines can be de novo methylated through the RdDM pathway. During canonical RdDM, SAWADEE HOMEODOMAIN HOMOLOGUE 1 is recruited to sites containing methyl groups on the ninth lysine of the histone 3 tail (H3K9) and directly interacts with and recruits Polymerase IV (Pol IV) to these sites initiating transcription of short ∼32 nt RNA transcripts. Pol IV then feeds this transcript directly into RNA-DEPENDENT RNA POLYMERASE 2 (RDR2) that then converts the single stranded RNA transcripts into double-stranded RNAs, which are then digested into 24 nt small interfering RNAs (siRNAs) by RNase III endonuclease DICER-LIKE 3 (DCL3), and loaded into ARGONAUTE 4, 6, or 9 (AGO4/6/9). Concurrently, a complex consisting of SU(VAR) homologs (SUVH) 2 or 9, DEFECTIVE IN MERISTEM SILENCING 3 (DMS3), DEFECTIVE IN RNA-DIRECTED DNA METHYLATION 1 (DRD1), RNA-DIRECTED DNA METHYLATION 1 (RDM1), and Pol V is brought to a locus through the interaction between DNA methylation and SUVH2/9. The RNA scaffold of Pol V transcripts can be recognized by the siRNA–AGO4/6/9 complex with help from SUPPRESSOR OF TY 5-LIKE (SPT5L). DOMAINS REARRANGED METHYLTRANSFERASE 2 (DRM2) is then recruited to sites recognized by sRNA-bound AGO4/6/9 through RDM1, allowing DRM2 to methylate the adjacent DNA. DRM2 adds methylation to the targeted DNA, SUVH4/5/6 binds the DNA methylation and deposits H3K9 methylation, which attracts the Pol IV arm of the pathway thus, creating a positive feedback loop that helps maintain the newly added methylation. siRNAs generated from sources other than Pol IV are sometimes incorporated into RdDM in a process called non-canonical RdDM. These siRNAs can be generated from inverted repeats, miRNA precursor’s, fragments of cleaved mRNA, or other non-coding dsRNAs that are processed by DCL proteins or a combination of AGO4 and exonucleases, loaded into AGO4/6/9, and directly recruit the POLV arm of the pathway to a target site. Removal of DNA methylation: DEMETER (DME) family of bifunctional glycosylase/lyases, consisting of DME and DME -LIKE 1/REPRESSOR OF SILENCING 1 (DML1/ROS1), DML2, and DML3, actively remove methylated cytosines which are then replaced with unmethylated cytosines by the base excision repair (BER) pathway. Created with BioRender.com.

Eukaryotic DNA is packaged into ∼146 base pair (bp) DNA segments that wrap around a histone octamer known as a nucleosome (Luger et al., 1997; Richmond and Davey, 2003; Ou et al., 2017). Nucleosomes are the base units of chromatin and can adopt two main configurations: euchromatin, which is less compact and more accessible to transcription factors and other proteins, or heterochromatin, which is more compact and less accessible (Roudier et al., 2009) (Figure 1). This means that gene regulation depends not only on the presence or absence of transcription factors, but also on chromatin accessibility (Li et al., 2007). Chromatin state can be altered by chromatin remodeling complexes (CRCs), histone modifications, histone variants, and DNA methylation, which work together to activate or repress of different transcriptional networks in eukaryotes (Li et al., 2007; Bannister and Kouzarides, 2011; Pikaard and Mittelsten Scheid, 2014; Zhong et al., 2021).

In Arabidopsis (*Arabidopsis thaliana*), CRCs, such as the SWITCHING DEFECTIVE 2/SUCROSE NON-FERMENTING 2 (SWI2/SNF2) proteins use ATP hydrolysis to alter the structure or positioning of nucleosomes, which in turn mediate the accessibility of the chromatin to transcription factors and other regulatory proteins (Corona and Tamkun, 2004; Jiang and Pugh, 2009). In addition to the CRCs, histone modifications and histone variants can affect transcriptional gene regulation by modulating histone–DNA interactions (Feng et al., 2010; Bannister and Kouzarides, 2011). The exposed N-terminal tails of the core histones are subjected to various post-translational modifications, including acetylation, methylation, phosphorylation, ubiquitylation, SUMOylation, etc. (Pfluger and Wagner, 2007). The addition or removal of these histone modifications corresponds with activation or repression of transcription (Feng et al., 2010; Bannister and Kouzarides, 2011). For example, adding acetylation to the histone tails via histone acetyltransferases is associated with transcriptional activation, while the removal of acetylation through histone deacetylases leads to transcriptional repression (Pandey et al., 2002; Lawrence et al., 2004).

Unlike histone acetylation, which corresponds with the activation of transcription, histone methylation can be associated with activation or repression of transcription, depending on which lysine residues are methylated (Xiao et al., 2016). For example, three methyl groups on the fourth lysine of the histone 3 tail (H3K4me3), H3K36me2, and H3K36me3 are associated with active transcription (Zhao et al., 2005; Xu et al., 2008; Zhang et al., 2009; Ding et al., 2012) (Figure 1). On the contrary, H3K27me1, H3K27me3, and H3K9me2 are associated with transcriptional repression (Goodrich et al., 1997; Gendall et al., 2001; Jackson et al., 2002; Malagnac et al., 2002; Johnson et al., 2007; Zhang et al., 2007; Bernatavichute et al., 2008; Jacob et al., 2009; Du et al., 2012).

DNA cytosine methylation, which in plants occurs in the CG, CHG, and CHH sequence contexts (H is any nucleotide other than G), has also been associated with gene regulation. Often, the presence or absence of DNA methylation is associated with repression and activation, respectively, of nearby genes or transposable elements. In plants, DNA methylation is maintained by four different DNA methylation maintenance pathways and can be established de novo by the RNA-directed DNA methylation (RdDM) pathway (Law and Jacobsen, 2010). The canonical RdDM pathway can be split into two different arms: the Polymerase IV (Pol IV) arm, which is responsible for the generation of small interfering RNAs (siRNAs), and the Pol V arm, which provides an RNA scaffold for the recruitment of the DOMAINS REARRANGED METHYLTRANSFERASE 2 (DRM2) at the target site (Erdmann and Picard, 2020) (Figure 1). While the majority of the siRNAs used in RdDM are produced by the Pol IV–RDR2 complex (Herr et al., 2005; Raja et al., 2008; Stroud et al., 2013; Huang et al., 2021), a small amount are generated from other sources including inverted repeats, microRNA (miRNA) precursors, fragments of cleaved mRNA, or other non-Pol IV generated non-coding RNAs that can also trigger a non-canonical RdDM pathway. These siRNAs are loaded into ARGONAUTE (AGO) proteins that direct the Pol V arm of the RdDM pathway to specific sites, and are important for pioneering sites of RdDM de novo (Allen et al., 2005; Slotkin et al., 2005; Vazquez et al., 2008; Chellappan et al., 2010; Khraiwesh et al., 2010; Wu et al., 2010, 2012; Garcia et al., 2012; Marí-Ordóñez et al., 2013; Nuthikattu et al., 2013; Creasey et al., 2014; Bond and Baulcombe, 2015; McCue et al., 2015; Panda et al., 2016; Yang et al., 2016; Ye et al., 2016; Sigman et al., 2021) (Figure 1).

DNA methylation is a mitotically and meiotically heritable mark (Law and Jacobsen, 2010). Maintenance of methylation by RdDM in euchromatic regions depends on siRNAs. DNA methylation in all sequence contexts is replicated on the newly formed daughter strands through the concerted effort of three additional DNA methyltransferases as well. Methylation at CG sites is maintained during replication by DNA METHYLTRANSFERASE 1 (MET1), through an interaction with VARIANT IN METHYLATION 1 (VIM1), VIM2, and VIM3 proteins that can bind to methylated cytosines in CG context on the parent strands (Woo et al., 2007, 2008). Non-CG methylation in heterochromatic regions is primarily maintained by the plant-specific CHROMOMETHYLASE 2 (CMT2) and CMT3 methyltransferases via a positive feedback loop with H3K9me2 histone modification and by RdDM in euchromatic regions (Lindroth et al., 2001; Du et al., 2012; Stroud et al., 2013, 2014; Erdmann and Picard, 2020). In plants, DNA methylation is actively removed by the DEMETER (DME) family of bifunctional glycosylase/lyases consisting of DME and DME -LIKE 1/REPRESSOR OF SILENCING 1 (DML1/ROS1), DML2, and DML3 (Choi et al., 2002; Gong et al., 2002; Agius et al., 2006; Gehring et al., 2006; Morales-Ruiz et al., 2006; Penterman et al., 2007; Ortega-Galisteo et al., 2008) (Figure 1).

## Technologies to recruit factors to manipulate transcription

Targeted manipulation of epigenetic marks or gene expression requires a way to specifically and ectopically recruit molecular components capable of transcriptional control or epi-mutagenesis to the site of interest on the genome. Several targeting technologies are reviewed in the following section.

## Small RNA-based targeting

**Figure 2:**
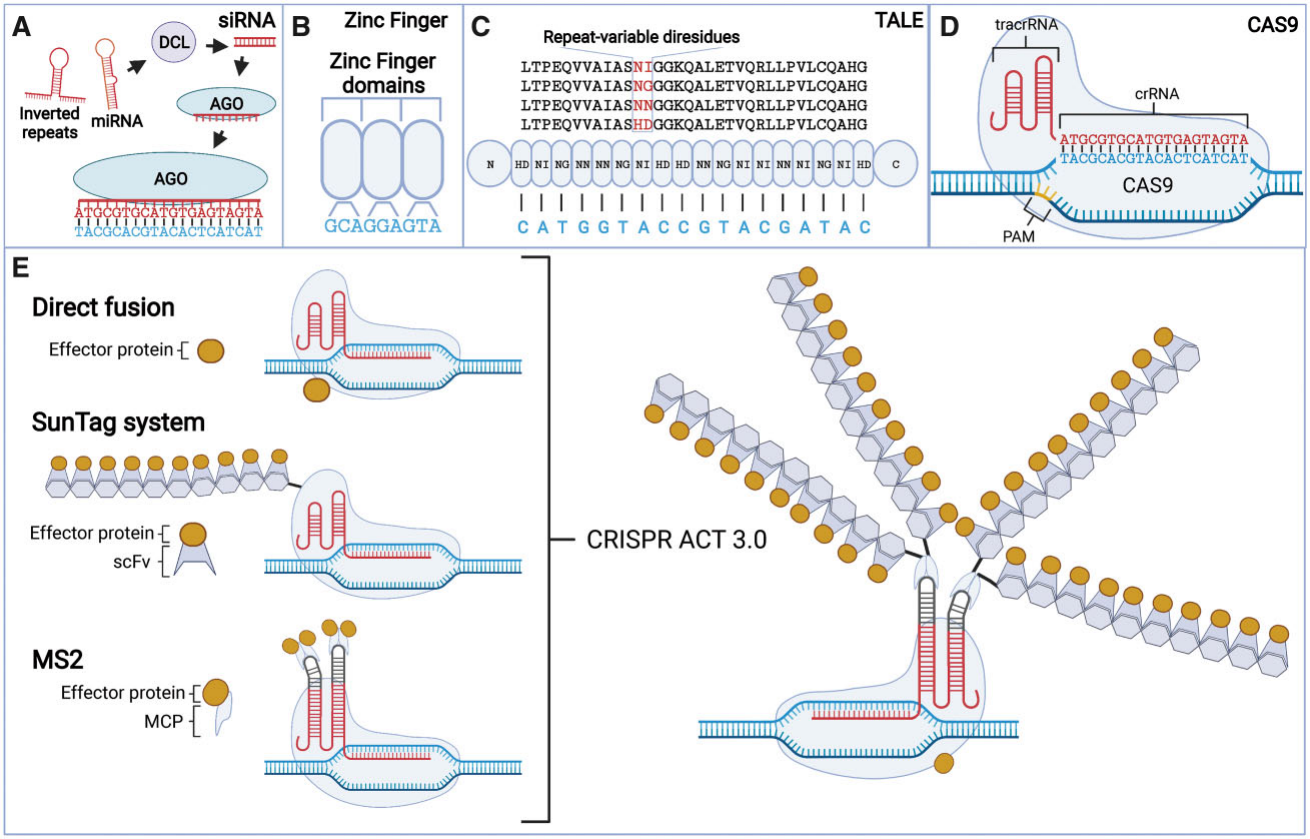
Targeting systems A. The small interfering RNA (siRNA) targeting system which takes advantage of artificially designed inverted repeats or microRNAs (miRNA) to target specific sequences through RNA-directed DNA methylation (RdDM) or RNA interference mechanisms. DICER-LIKE (DCL); ARGONAUTE (AGO) B, The Zinc Finger targeting system where each artificially designed Zinc Finger domain is capable of recognizing a unique nucleotide triplet. C, The TRANSCRIPTION ACTIVATOR-LIKE EFFECTOR (TALE) targeting system highlighting the RVDs, which give nucleotide binding specificity to each repeat unit. D, The CRISPR–Cas9 targeting system highlighting the CRISPR RNA (crRNA), trans-acting crRNA (tracrRNA) and protospacer adjacent motif (PAM). E, Direct fusion, SunTag, and MS2 based CRISPR–dCas9 systems and how these have been combined in the CRISPR ACT 3.0 system. MS2 coat protein (MCP), Single chain variable fragment (ScFV). Created with BioRender.com.

Both siRNAs and miRNAs have been broadly applied for transcriptional and post-transcriptional gene silencing, not only because small RNA-mediated gene silencing is sequence specific and efficient, but also because they can cause partial loss-of-function alleles that can overcome the lethality of certain null mutants (Ossowski et al., 2008). Endogenously, siRNAs and miRNAs are the slicing products of DICER-LIKE (DCL) proteins from double-stranded RNAs (dsRNAs) and primary miRNA transcripts with a stem–loop structure, respectively (Kurihara and Watanabe, 2004; Xie et al., 2005) (Figure 2A). Both siRNAs and miRNAs are loaded into AGO proteins to form an RNA-induced silencing complex (Mallory and Vaucheret, 2010), which in turn silences the target gene with the complementary sequence (Huang et al., 2019). One of the most popular methods for the generation of synthetic siRNAs or miRNAs in plants is to express hairpin RNA or inverted repeats that are linked by an intron sequence. This hairpin RNA can be recognized and processed by plant DCLs into siRNAs and then cause the target gene silencing (Chuang and Meyerowitz, 2000; Wesley et al., 2001). While these are frequently designed to target exon regions to trigger post-transcriptional gene silencing, they can also be designed to target promoter regions to trigger RdDM-directed silencing (Mette et al., 2000; Dadami et al., 2014; Williams et al., 2015; Gallego-Bartolomé et al., 2019). Some other small RNA-mediated approaches to silence genes include virus-induced gene silencing (Wassenegger et al., 1994; Baulcombe, 1999; Burch-Smith et al., 2004), and artificial miRNA directed gene silencing (Schwab et al., 2006; Ossowski et al., 2008).

While synthetic siRNAs and miRNAs can target gene silencing, they cannot recruit other factors or be used for targeted activation, and are prone to off-target effects (Xu et al., 2006; Ossowski et al., 2008) (Table 1). Technologies allowing for the targeting of effector domains in a sequence dependent manner allow for more specific control of transcriptional activation in addition to repression.

**Table 1:** Comparison of targeting systems

Parameters	sRNA	TALE	Zinc Finger	CRISPR–dCas9
1. Target sites	No limit	Occurs every ∼35 bp	Occurs at every ∼200–500 bp	Depends on PAM sites. New PAM-less CRISPR variants are available that increase available target sites
2. Specificity	Less specific	More specific than other technologies	Less specific	Highly specific
3. User friendly	Easy to clone constructs	Cloning is laborious and tedious	Cloning is laborious and tedious	Easy to clone constructs
*a. Cloning*				
*b. Adaptability to target new sites*	Highly adaptable— only need to modify the precursor RNA sequence	Less adaptable—must design new protein for each new target; protein engineering can be unpredictable	Less adaptable—must design new protein for each new target; protein engineering can be unpredictable	Highly adaptable—only need to modify the guide RNA sequence
*c. Cost*	Targeting new site depends on manipulating the precursor sequence, which is less expensive	Can be expensive as it requires testing of several TALEs to target new site	Can be expensive as it requires testing of several Zinc Fingers to target new site	Targeting new site depends on manipulating guide RNA sequence, which is less expensive

## Sequence-dependent DNA binding modules

### Zinc Fingers

Zinc Fingers (ZFs) are one of the earliest and best characterized tools for targeting effector domains to specific regions of a genome. These proteins typically contain a classical C2H2 ZF structure with two β-sheets and one α-helix maintained by hydrophobic interactions and a zinc ion (Lee et al., 1989). Each finger primarily recognizes and binds to a unique 3-bp DNA sequence encoded in the amino acid residues of the α-helix (Pavletich and Pabo, 1991; Elrod-Erickson et al., 1996) (Figure 2B). This amino acid sequence can be manipulated and repeated to develop artificial ZF proteins with multiple finger domains capable of differentiating the DNA sequence of a target site from the rest of the genome (Liu et al., 1997; Segal et al., 1999; Dreier et al., 2001). These artificial ZFs can then be fused to an effector domain in order to activate or repress transcription. While ZF targeting systems have been widely used, they have several drawbacks relative to other targeting systems, such as a relative lack of specificity, which are outlined in Table 1 (Beerli et al., 1998; Johnson et al., 2014; Gallego-Bartolomé et al., 2019; Gardiner et al., 2020; Liu et al., 2021).

### Transcription activator-like effectors

Like ZFs, TRANSCRIPTION ACTIVATOR-LIKE EFFECTORS (TALEs) contain specific amino acid sequences that allow for the programmable recognition of specific DNA sequences (Figure 2C). The TALE DNA binding domain consists of around 18 units of repeats with each unit comprising ∼34 amino acids containing two variable amino acids at positions 12 and 13, known as repeat-variable diresidues (RVDs), which direct the binding specificity of a unit (Boch et al., 2009; Moscou and Bogdanove, 2009) (Figure 2C). Thus, by rearranging these repeat units, designer TALEs can be created to target specific sequences (Boch et al., 2009; Morbitzer et al., 2010). Assembly of additional TALEs to target a unique site is mostly a matter of assembling these repeat units so that the RVD nucleotide preference matches the target site. Certain RVDs either only bind to or are unable to bind to methylated DNA (Bultmann et al., 2012; Deng et al., 2012; Valton et al., 2012; Tsuji et al., 2016), and can be used to build TALE constructs that can discriminate between methylated and unmethylated recognition sites (Deng et al., 2012; Valton et al., 2012; Tsuji et al., 2016). While a powerful tool, TALEs are difficult to assemble due to the repetitiveness of the units, which only differ by two amino acids (Cermak et al., 2011; Morbitzer et al., 2011; Zhang et al., 2011) (Table 1).

### CRISPR–Cas direct fusion

CRISPR**–**Cas systems can be used to target specific regions of a genome. These systems are naturally occurring in bacteria and archaea, and evolved as a type of adaptive immune system (Sorek et al., 2013). Unlike ZF or TALE systems that use the manipulation of amino acid sequences to specify the target site, CRISPR**–**Cas systems use non-coding RNAs (Figure 2D). The natural targeting system consists of an RNA sequence complementary to the target sequence known as the spacer or CRISPR RNA (crRNA) and a scaffold sequence that is bound by the Cas protein known as the trans-activating crRNA (tracrRNA) (Deltcheva et al., 2011) (Figure 2D). To simplify the tool, the crRNA and tracrRNA have been fused to form a single RNA molecule called a guide RNA (gRNA) (Jinek et al., 2012). The gRNA component is loaded into a Cas protein that is then able to identify and create a double-stranded break at the appropriate target site. Recognition of the target sequence also requires a protospacer adjacent motif (PAM) specific to the Cas protein being used limiting the number of possible targets (Figure 2D). However, recent advancements in the development of CRISPR–Cas systems have created systems that do not require this PAM motif (Walton et al., 2020; Ren et al., 2021). In addition to using Cas to trigger mutations at target sites, modified systems using catalytically inactive Cas (dCas) fused to an effector domain can be designed to cause a wide range of targeted effects, depending on the effector domain used (Jinek et al., 2012; Larson et al., 2013; Qi et al., 2013; Lowder et al., 2015; Piatek et al., 2015; Liu et al., 2016; Li et al., 2017, 2020; Tang et al., 2017; Khakhar et al., 2018; Selma et al., 2019; Ghoshal et al., 2021).

The reduced expense, ease of construction, and high specificity of targeting has made CRISPR–dCas systems incredibly popular leading to the further development of more advanced systems beyond the simple direct fusion. To enhance the targeted effect of these systems, additional systems with the ability to synergistically target multiple effector domains to a single locus have been developed as discussed below.

### SunTag

The SunTag system was originally developed in animals to recruit multiple copies of GREEN FLUORESCENT PROTEIN (GFP) to a single locus allowing for the visualization of the target (Tanenbaum et al., 2014). This was quickly incorporated into a dCas9-based system for other uses such as the activation of transcription or the addition/removal of DNA methylation in both plants and animals (Tanenbaum et al., 2014; Morita et al., 2016; Gallego-Bartolomé et al., 2018; Pflueger et al., 2018; Papikian et al., 2019; Tang et al., 2021). The SunTag-dCas9 system requires the coordinated expression of three different components: a dCas9 fused to a peptide tail containing an array of epitope repeats; a complementary single-chain variable fragment (scFv) fused to an effector domain and a gRNA (Figure 2E). This enables each dCas9 to recruit multiple copies of the effector domain via interactions between the scFv and the epitope tail. While this increases the complexity of the system, recruiting multiple copies of an effector domain to a single locus has proven more effective than targeting a single effector domain through a direct fusion to dCas9 (Morita et al., 2016; Pflueger et al., 2018). More recently, epitope tails containing a combination of two different epitopes bound by separate scFvs have enabled the co-targeting of two different unique scFv-effector fusions at the same time (Boersma et al., 2019). Recruiting multiple copies of an effector protein using the SunTag system raises the possibility that these systems can have a larger targeted epigenetic footprint than the directly fused dCas9-effector version. Although this might be true in certain cases, the targeted epigenetic footprint seems to be influenced by several factors. For example, in mammalian cell lines, targeting the mammalian de novo DNA methyltransferase DNA-methyltransferase 3 alpha (DNMT3A), fused directly to dCas9 resulted in a DNA methylation footprint of approximately 200 bp (McDonald et al., 2016; Vojta et al., 2016). While using the SunTag system with the DNMT3A resulted in a wider DNA methylation footprint of ∼4 kb on the *Homeobox A5* (*HOXA5*) gene, this was not observed when the DNMT3A-SunTag system targeted the *Krüppel-like factor 4* gene, indicating a locus-specific effect (Huang et al., 2017). The influence of genomic context was also implied in plants (Ghoshal et al., 2021). For example, in Arabidopsis, identical targeted DNA methylation footprints were observed at the *FWA* gene when targeting a CG-specific bacterial methyltransferase using either a direct fusion with dCas9 or the SunTag system. This was most likely due to the targeted region being flanked by genomic regions lacking CG sites limiting the span of DNA methylation targeted by both tools. Thus, further research is required to directly assess the factors influencing the DNA methylation footprints induced by these multi-effector protein targeting tools.

### MS2

Like the SunTag system, the MS2 system is a CRISPR–dCas9-based system that gives the user the ability to target multiple effector proteins to a specific locus (Konermann et al., 2015). However, unlike SunTag or direct fusions to dCas9, the MS2 system recruits effector proteins via interactions with a modified gRNA. The MS2 system takes advantage of the MS2 bacteriophage coat protein and its known RNA binding site. Like the SunTag system, this system also requires the coordinated expression of three components: a dCas9, a MS2 coat protein-effector domain fusion, and a gRNA scaffold including an MS2 binding site added to the tetraloop and/or stem loop 2 positions of the tracrRNA (Konermann et al., 2015) (Figure 2E). The MS2 system can be combined with dCas9 direct fusion (known as the Synergistic Activation Mediator system or SAM) or the SunTag system or both (known as CRISPR Act 3.0) to recruit even more or different effector proteins to a target site via effector protein interactions with both the gRNA and the dCas9 (Konermann et al., 2015; Lowder et al., 2018; Pan et al., 2021) (Figure 2E).

## Targeted epi-mutagenesis and transcriptional control

### Effector domains used for transcriptional activation

There are an array of different effector domains that can be used to activate transcription in targeted systems. This can be achieved directly by attracting basal transcription factors, or indirectly by adding active histone marks or removing repressive marks. Targeting of the modular activating domain of the herpes simplex virus VP16 to a specific locus has been extensively described as an efficient way to activate transcription (Dreier et al., 2001; Sánchez et al., 2002; Stege et al., 2002; Morbitzer et al., 2010; Geißler et al., 2011; Miller et al., 2011; Zhang et al., 2011; Cheng et al., 2013; Maeder et al., 2013; Mali et al., 2013; Perez-Pinera et al., 2013; Qi et al., 2013; Liu et al., 2014; Tanenbaum et al., 2014; Vazquez-Vilar et al., 2016; Li et al., 2017; Park et al., 2017; Lee et al., 2019; Papikian et al., 2019; Selma et al., 2019).The VP16 activator domain is an acidic peptide that interacts with basal transcription factors and the mediator complex to facilitate the assembly of the pre-initiation complex at its target site (Hall and Struhl, 2002; Hirai et al., 2010). Vp16 also interacts with histone acetyltransferases and the SWI/SNF ATPase complex to manipulate the surrounding chromatin structure into an active state (Hall and Struhl, 2002; Hirai et al., 2010). Creating a tetramer (VP64) or octamer (VP128) of the minimal VP16 activator domain can dramatically increase the potency of this activator (Beerli et al., 1998; Li et al., 2017). In addition to this, recruiting multiple copies of VP64 via systems like the SunTag can improve the activation of downstream targets compared to direct fusion systems (Tanenbaum et al., 2014; Lowder et al., 2018; Selma et al., 2019).

Directly targeting transcriptional activation can also be achieved through targeting the highly conserved plant-specific acidic-type activator domains found in the APETALA2 family of proteins, known as the EDLL domains. The EDLL domain of AtEFR98 is frequently used as a modular component to activate gene expression (Tiwari et al., 2012; Piatek et al., 2015; Selma et al., 2019). This EDLL motif is relatively small (24 amino acids) compared to other common activator domains like the VP16 (78 amino acids) making this activating domain an attractive option for development of compact synthetic targeted activation systems. However, at some target sites, multiple copies of EDLL were needed to achieve similar transcriptional activation as VP16 (Tiwari et al., 2012). Like VP16 and EDLL domains, TAL acidic-type activator domains, found in natural TALE systems, have also been used in a modular way to activate gene expression (Piatek et al., 2015; Li et al., 2017; Selma et al., 2019). This effector domain, when targeted using the TALE system, can activate genes upstream and downstream of its binding site regardless of which strand the effector is targeted to (Wang et al., 2017).

In addition to the direct activation of transcription by recruiting activator domains, another option is recruiting domains capable of adding active or removing repressive epigenetic marks, thereby activating gene expression indirectly. In plants, targeting transcriptional activation through H3K27 acetylation using the p300 domain from humans or the catalytic domain of the plant-specific ARABIDOPSIS HISTONE ACETYLTRANSFERASE OF THE CBP FAMILY 1 (HAC1) can activate transcription of targeted genes; however, at least in the case of p300, a higher level of activation is achieved when VP64 is used (Lee et al., 2019; Roca Paixão et al., 2019; Selma et al., 2019). Removing repressive DNA methylation from a promoter using the human TEN-ELEVEN TRANSLOCATION1 (TET1) can also cause transcriptional activation (Maeder et al., 2013; Amabile et al., 2016; Choudhury et al., 2016; Liu et al., 2016; Morita et al., 2016; Xu et al., 2016; Lo et al., 2017; Okada et al., 2017; Gallego-Bartolomé et al., 2018; Li et al., 2020; Tang et al., 2021). In animals, the TET family of oxidases can oxidize methylated DNA, leading to either the passive removal of the methyl group through a lack of maintenance during DNA replication or the active removal by glycosylases such as thymine DNA glycosylase (Lio et al., 2020). Such oxidized variants have been detected in plants; however, at such low levels that its importance is called into question (Mahmood and Dunwell, 2019). While no proteins homologous to the TET family have been described in plants, ectopic expression of TET proteins, targeted or otherwise, in plants causes a loss of DNA methylation, suggesting the existence of a similar passive or active mechanism for the removal of oxidized DNA methyl groups in plants (Hollwey et al., 2016; Gallego-Bartolomé et al., 2018;  Ji et al., 2018).

Unlike recruiting the basal transcriptional machinery, which can cause unwanted overexpression, using epigenetic marks to control transcription only facilitates the accessibility of the target promoter to the transcriptional machinery. This highlights an advantage to the manipulation of epigenetic marks over the targeting of an activator domain. In addition to this, altering epigenetic marks can also facilitate the accessibility of targeted activator domains and thus, achieve a synergistic effect when they are combined (Roca Paixão et al., 2019).

### Co-targeting Activation

While many studies have attempted to compare single activator domains to determine the best one to use for targeted activation, such studies will be heavily influenced by a number of unrelated factors, including the choice of targeting system and target site. While an activator domain might appear superior in a specific system or at a specific target site, this cannot be extrapolated to every possible scenario that exists in every plant genome, and thus a variety of tools are needed. However, a consistent trend seen in the development of these tools is that the ones that are capable of targeting multiple activator domains to a single locus have consistently been shown to perform better than those targeting a single domain (Beerli et al., 1998; Tiwari et al., 2012; Li et al., 2017; Selma et al., 2019; Morita et al., 2020; Pan et al., 2021). While there are limits to the number of domain repeats that can be included in a single coding sequence due to protein instability, using multiple different activator domains to synergistically activate gene expression has been an immensely successful strategy (Li et al., 2017; Selma et al., 2019). Targeting direct fusions of VP128 to EDLL or VP128 to TAL activator domains leads to an increase in the activation of gene expression (Li et al., 2017). In addition the fusion of VP64 to P65 and Rta, two additional modular activator domains originally shown to work in animals (VPR), is also capable of activating gene expression in plants to a higher level than VP64 alone (Chavez et al., 2015; Li et al., 2017). Further, systems that allow the co-targeting of different activator domains can now push this further by combining even more activator domains such as the EDLL domain with the VPR fusion (Selma et al., 2019). However, quantitatively comparing these different co-targeting strategies is challenging and again depends on the target site and targeting system. For example, co-targeting the EDLL domain with multimers of the VP16 activator domain either through the SAM targeting system or direct fusion, has been shown to be inferior to fusions to other activator domains or simply by co-targeting multiple VP64 peptides to the target locus (Li et al., 2017; Lowder et al., 2018; Pan et al., 2021). However, when the EDLL domain is co-targeted along with the VPR fusion using the SAM targeting system, it has been shown to produce a higher gene activation than targeting multiple VP64 domains. Thus, if an activator domain is effective at one locus or with a particular targeting system, it does not mean it will be effective when used in a different system or at a different locus.

Further, in animal cells, when VP64 and TET1 were co-targeted to the same locus they worked synergistically to upregulate gene expression causing a greater fold change increase in expression than targeting either factor alone (Morita et al., 2020), suggesting that manipulating the epigenetic landscape to be more amenable to activation is synergistic with targeting activator domains. In plants, targeting multiple VP64 activator modules to the *FWA* promoter using the SunTag targeting system also resulted in the loss of DNA methylation, suggesting that simultaneously targeting mechanisms that remove DNA methylation and directly activate of gene expression could work synergistically in plants as it does in animals (Papikian et al., 2019; Morita et al., 2020).

### Effector domains used for transcriptional repression

Targeted transcriptional repression can be achieved by several mechanisms such as chromatin remodeling, adding repressive or removing active epigenetic marks on histones, adding DNA methylation, inhibiting RNA Polymerase II processivity, or by triggering degradation of mRNA.

The most widely used effector domains for targeted gene repression in plants are the ERF-associated amphiphilic repression (EAR) domains, such as those found in SUPERMAN or BODENLOS. Outside of the targeting systems discussed here, these domains have been used extensively as a way to study highly redundant genes, as these repressor domains are dominant over activator domains, including VP16, and can be used to turn a constitutive activator into a constitutive repressor (Hiratsu et al., 2002, 2004; Kagale and Rozwadowski, 2011). EAR domains have been found to recruit histone deacetylases such as HDA19 and co-repressors such as TOPLESS (Kagale and Rozwadowski, 2011). The EAR domain SUPERMAN-REPRESSIVE DOMAIN X, an optimized version of the SUPERMAN EAR domain, and the downstream co-repressor TOPLESS have been used together with the targeting systems discussed above to specifically target transcriptional repression (Lowder et al., 2015; Tang et al., 2017; Khakhar et al., 2018).

Targeted addition of DNA methylation to promoters using DNA methyltransferases has also been used successfully to repress transcription. The catalytic domain of the RdDM-based DNA methyltransferase from tobacco (*Nicotiana tabacum*), DOMAINS REARRANGED METHYLTRANSFERASE (DRM) can add DNA methylation to a target promoter, resulting in transcriptional repression (Papikian et al., 2019). The inheritance of the targeted DNA methylation and thus, the transcriptional repression is highly dependent on the levels of CG methylation established at the target site (Gallego-Bartolomé et al., 2019). To increase the heritability of the targeted methylation recent studies in plants have used the CG-specific bacterial *Mollicutes Spiroplasma* DNA methyltransferase MQ1 containing a Q147L mutation to increase the specificity of the effector domain (Ghoshal et al., 2021). In the absence of the RdDM pathway, the targeted gene was found to only have CG methylation, while in wild type plants non-CG methylation was also observed, indicating that MQ1 only installs CG methylation to the target site which then recruits other DNA methylation (Ghoshal et al., 2021).

Besides modifying the epigenome for transcriptional repression, a direct mechanism to repress gene expression is by hindering the movement of the RNA polymerase II, known as CRISPR interference (CRISPRi). This is achieved by targeting CRISPR–dCas-based systems to the TSS region or downstream of the transcriptional start site of a gene and has been well demonstrated in mammalian cells (Qi et al., 2013). However, reports of CRISPRi are limited in plants. Only one example has shown partial repression of a gene by CRISPRi in maize (*Zea mays*) (Gentzel et al., 2020). In plants, no studies have been reported on co-targeting different repressor domains to repress transcription. However, in animals, recruiting a DNMT3a domain, a DNMT3-LIKE (DNMT3L) domain, and a Krüppel-associated box transcriptional repression domain to a target gene via direct fusion of all three to dCas9 has been demonstrated as a powerful gene repression strategy (Nuñez et al., 2021).

## Conclusions

While a number of platforms have been developed for targeted transcriptional control and epi-mutagenesis in plants, including small RNAs, ZFs, and TALEs, development of CRISPR-based systems have recently become the dominant focus due to their ease of use and flexibility. Recent developments in CRISPR–dCas systems have focused on increasing efficiency and functionality, including creating systems capable of recruiting multiple copies and multiple types of effectors to a target site. In addition to this, progress has also been made in the development of additional and optimized delivery systems for these tools (Box 1). The development of CRISPR–dCas systems for targeted transcriptional control and epi-mutagenesis is still in its infancy and there are many ways in which these tools can be improved (see Outstanding Questions). Their recent development means that these tools have only been utilized in a few studies outside of the ones creating or optimizing them; however, examples are available and demonstrate the usefulness of these systems in answering basic questions and bioengineering (Lee et al., 2021; Leydon et al., 2021). The further development of these tools provides us with additional ways to target specific transcriptional or epigenetic manipulations in plants, allowing us to collect more direct evidence for the function of epigenetic marks and genes which can then be applied to the benefit of agriculture.

ADVANCES

- Recent advancements in DNA targeting systems have largely focused on the development of CRISPR–dCas systems to include peptide tails and RNA binding proteins, allowing these systems to recruit multiple copies of any given effector protein.
- Effector protein domains capable of manipulating DNA methylation have been successfully deployed and, in some cases, changes have been shown to be heritable to the next generation in the absence of the targeting construct.
- Fusion and co-targeting strategies using common modular activator domains can synergistically activate transcription to levels higher than targeting with a single type of activator domain.
- CRISPR-dCas systems can be used to repress genes by targeting of modular repression domains, DNA methylation, or CRISPRi.

OUTSTANDING QUESTIONS

- What effector domains can be used synergistically through direct fusion or co-targeting to create artificial protein complexes to regulate the chromatin environment through histone marks, DNA methylation, and basal transcription factors to ensure heritability of the transcriptional modification, allowing the targeting construct to be segregated out in later generations without reversion?
- Can tissue-specific expression of advanced CRISPR–dCas systems improve targeting efficiency, as has been the case with ZF and CRISPR–Cas9 constructs?
- How can we create tools which allow for the targeting of different effectors to separate sites in the genome to provide the ability to adjust entire transcriptional networks by activating and repressing specific components simultaneously?
- Can these tools be delivered to plants using a non-transgenic approach?

BOX 1 Advances in the delivery of targeting systems Generally, the preferred mode of delivery for CRISPR–Cas systems in plants has been through generating stable transgenic plants by *Agrobacterium tumefaciens*-mediated gene transfer or particle bombardment (Altpeter et al., 2016; Lowder et al., 2017, 2018; Tang et al., 2017; Gallego-Bartolomé et al., 2018; Lee et al., 2019; Papikian et al., 2019; Ghoshal and Gardiner, 2021; Ghoshal et al., 2021). However, transient delivery methods provide the advantage of spatially and temporally regulating the expression of the CRISPR–Cas systems in the plants. This can reduce the off-target effects of CRISPR–Cas systems and bypass the process of generating transgenic plants, which can be tedious and time-consuming for many crop species (Altpeter et al., 2016). Transient methods such as agroinfiltration, viral-based delivery approaches, lipid-based systems, or nanomaterial-based techniques have been used for the transient delivery of genome editing systems into plants (Sandhya et al., 2020). So far, only agroinfiltration and viral delivery approaches have been adopted to transiently deliver CRISPR components for regulating gene expression or for epimutagenesis in whole plants (Piatek et al., 2015; Ghoshal et al., 2020; Khakhar and Voytas, 2021). Delivery of dCas-based reagents by PEG-mediated transformation for transcriptional activation has only been achieved in protoplasts (Li et al., 2017; Lowder et al., 2018). Viral delivery of CRISPR reagents to whole plants has excellent potential for developing non-transgenic approaches for genome and epigenome editing; however, it has two major challenges. The first is ensuring the efficient delivery of the CRISPR reagents to the appropriate targeted cells, such as meristematic cells, that facilitate the inheritance of the introduced change (Altpeter et al., 2016). Recent studies have used plant mobile RNA signal sequences that enhance the movement of the RNA viruses to facilitate the delivery of CRISPR reagents into meristematic cells (Ghoshal et al., 2020; Ma et al., 2020; Zhang et al., 2020; Khakhar and Voytas, 2021). The second challenge is the limited cargo size of viral vectors. For genome editing, studies with certain viruses have shown to be promising to deliver large cargoes, such as the whole of CRISPR–Cas9 constructs (Zhang et al., 2020). However, these viruses have a limited host range and such trials remain to be explored for epigenome editing purposes, so additional strategies are required to deliver CRISPR–dCas9 components efficiently.

## Notes

All authors participated in writing of the article.

The author responsible for distribution of materials integral to the findings presented in this article in accordance with the policy described in the Instructions for Authors (https://academic.oup.com/plphys/pages/general-instructions) is Steven E. Jacobsen (jacobsen@ucla.edu).
